# Integrating SANS and fluid-invasion methods to characterize pore structure of typical American shale oil reservoirs

**DOI:** 10.1038/s41598-017-15362-0

**Published:** 2017-11-13

**Authors:** Jianhua Zhao, Zhijun Jin, Qinhong Hu, Zhenkui Jin, Troy. J. Barber, Yuxiang Zhang, Markus Bleuel

**Affiliations:** 1 School of Geosciences, China University of Petroleum (East China), Qingdao, 266580 China; 2 State Key Laboratory of Shale Oil and Gas Enrichment Mechanisms and Effective Development, Beijing, 100083 China; 3 Petroleum Exploration and Production Research Institute, SINOPEC, Beijing, 100083 China; 4 Department of Earth and Environmental Sciences, the University of Texas at Arlington, Arlington, TX 76019 USA; 5 College of Geosciences, China University of Petroleum, Beijing, 102249 China; 6 NIST Center for Neutron Research, National Institute of Standards and Technology, Gaithersburg, MD 20899 USA; 7 Department of Materials Science and Engineering University of Maryland, College Park, MD, 20742-2115 USA

## Abstract

An integration of small-angle neutron scattering (SANS), low-pressure N_2_ physisorption (LPNP), and mercury injection capillary pressure (MICP) methods was employed to study the pore structure of four oil shale samples from leading Niobrara, Wolfcamp, Bakken, and Utica Formations in USA. Porosity values obtained from SANS are higher than those from two fluid-invasion methods, due to the ability of neutrons to probe pore spaces inaccessible to N_2_ and mercury. However, SANS and LPNP methods exhibit a similar pore-size distribution, and both methods (in measuring total pore volume) show different results of porosity and pore-size distribution obtained from the MICP method (quantifying pore throats). Multi-scale (five pore-diameter intervals) inaccessible porosity to N_2_ was determined using SANS and LPNP data. Overall, a large value of inaccessible porosity occurs at pore diameters <10 nm, which we attribute to low connectivity of organic matter-hosted and clay-associated pores in these shales. While each method probes a unique aspect of complex pore structure of shale, the discrepancy between pore structure results from different methods is explained with respect to their difference in measurable ranges of pore diameter, pore space, pore type, sample size and associated pore connectivity, as well as theoretical base and interpretation.

## Introduction

The continual increase of shale gas and tight oil production in the United States has changed the energy landscape, and led to a great interest in the exploration and production of shale oil and gas worldwide. The U.S. Energy Information Administration (EIA) estimates that about 13.5 trillion cubic feet of dry natural gas and about 4.9 million barrels per day of crude oil were directly produced from shale resources in the United States in 2015^1^. Both shale gas and tight oil productions account for about 50% of total U.S. dry natural gas and crude oil production in 2015^1^.

Although shale reservoirs contain a great resource potential, its complexity and heterogeneity of pore systems directly impact reservoir quality and production behavior^2–7^. Recently, the attributes of dominant nano-scale pore systems have been widely studied with respect to pore type, specific surface area, pore-size distribution, and connectivity, which control the storage of various oil/gas states (free, adsorption and dissolution) and fluid properties in shale^8–17^. Many experimental techniques have been used to characterize pore systems in shale, and they include direct high-resolution imaging methods [such as SEM (scanning electron microscopy), CT-scan, focused ion beam-SEM, helium ion microscopy and transmission electron microscopy^10,18–22^] as well as indirect methods [such as low-pressure gas physisorption^23–25^, mercury intrusion capillary pressure (MICP)^5,26,27^, and small-angle neutron scattering (SANS) and ultra-small-angle neutron scattering (USANS)^3,28–31^].

More importantly, different techniques are based on different experimental conditions, principles and data interpretation approaches; therefore, some differences of shale pore structure are commonly observed. It is particularly challenging to obtain the results over a wide scale without modifying the original pore structure information of shale. Furthermore, due to minimal contribution to hydrocarbon production, closed pores are often ignored in conventional oil and gas reservoirs. However, closed pores can store an appreciable amount of oil and gas, especially within organic matter particles in unconventional shale reservoirs, which could be released after hydraulic fracturing^31^. Therefore, closed porosity is also an essential factor to control oil/gas storage, transport pathway, and production behavior^23,28,32,33^. A clear understanding of the fundamental characteristics of pore structure, especially an assessment of the presence and contribution of closed pores, is crucial to sustainable shale oil/gas development.

Combined SANS and USANS methods have been reported to obtain a wide pore size distribution for coals, siltstones, carbonates, and shales under the conditions of minimal alteration of their original pore structure^2,3,29,34–36^. Compared with fluid-invasion approaches such as gas physisorption and MICP, which only measure connected pores accessible from the sample surface, (U)SANS method is a powerful tool to effectively characterize the total porosity (i.e. accessible and inaccessible pores) over a wider size range. Therefore, an integrated use of SANS and fluid-invasion techniques can distinguish accessible and inaccessible pores and provide a feasible approach to calculating closed porosity^19,23,28,37^. Nano-CT is another method capable of examining closed pores; however, it has a limitation of a voxel size of 50 nm for a sample size of only 65 μm^38^.In this study, the pore structure of four samples from typical American shale oil reservoirs (Niobrara, Wolfcamp, Bakken and Utica Formations; in the order of increasing geological age) was investigated using SANS, low-pressure nitrogen physisorption (LPNP), and MICP methods. The main purposes of this work are to (1) interpret porosity, pore-size distribution, and multiple-scale inaccessible porosity through multiple methods; and (2) compare the results for different oil shales and discuss the implications of different methods in studying pore structure of shale. The research will provide an improved understanding of the pore structure of shale reservoirs and a theoretical basis for the prediction of favorable shale oil reservoir.

## Methods

### Sample description

In this study, four core samples of different age, mineral composition, TOC (total organic carbon) content and thermal maturity were taken in four target strata of shale oil reservoirs (Table 1). The samples include (1) Niobrara marl form the 3 Berthoud State well, between chalk sections C and D in Montezuma County of Colorado^39^; (2) Wolfcamp shale of the upper Wolfcamp Formation from an undisclosed well in the Midland Basin, Texas; (3) Upper Bakken shale from the Kubas 11–13TFH well in Stark County of North Dakota; and (4) Utica shale from the Fred Barth #3 well in Coshocton County of Ohio.

**Table 1:** Parameters used in the analysis of the studied samples.

Sample ID	Age	Depth (m)	TOC (%)	T_max_	Calculated R_o_ (%)^*^	MICP		SANS			
				(°C)		bulk density (g/cm^3^)	grain density (g/cm^3^)	SLD ( × 10^−10^ cm^−2^)	Background (cm^−1^)	Slope	Fractal dimension
Niobrara	Cretaceous	939.0	2.88	442	0.79	2.45	2.56	4.21	0.43	3.5	2.5
Wolfcamp	Permain	/	3.40	450	0.94	2.56	2.57	3.71	0.09	3.2	2.8
Bakken	Devonian to Mississippian.	3238.8	9.79	435	0.67	2.44	2.48	2.66	0.18	3.1	2.9
Utica	Ordovician	1719.7	3.60	456	1.05	2.58	2.60	3.92	0.20	3.1	2.9

*Calculated R_o_ is obtained from pyrolysis-derived T_max_ values using the equation (T_max_ = (%R_o_ + 7.16)/0.0180) reported by Jarvie *et al*. (2001)^98^.

The samples were prepared by dry-cutting core plugs (25 mm in diameter and about 60 mm in length) parallel to the bedding plane to obtain thin sections polished to 150 μm in thicknesses, and glued onto quartz slides for SANS tests^19^. Other parts of core plugs were processed as different bulk and granular sample sizes for MICP (1 cm sided cube), LPNP (500–850 μm), X-ray diffraction XRD (<75 μm), LECO TOC (<75 μm), and pyrolysis/thermal maturity (<75 μm) tests.

### SANS experiments

SANS tests were conducted at the National Institute of Standards and Technology Center for Neutron Research (NIST-NCNR) using the NG7 30 m SANS instrument^19,40^. The scattering vector, or momentum transfer, of a scattered neutron is defined by Q = 4πλ−1 sin (θ/2), where θ is the scattering angle and λ is the wavelength of the monochromatic neutron beam. To obtain a wide scattering vector range of 0.001−0.28 Å^−1^, three sample-detector distances (1 m, 4 m, 13 m and 13 m) were used with neutron wavelengths of 6.0 Å (1, 4, and 13 m) and 8.09 Å (13 m lens). In order to reduce multiple scattering, specimens were mounted on quartz glass slides and ground to 150 µm thickness, such that a high neutron transmission and less than 10% multiple scattering are achieved^19^. Raw, 2D data were corrected for detector pixel efficiency, background and empty-cell scattering, as well as sample neutron transmission and volume. Corrected scattering intensities at each detector geometry are normalized to the intensity of the open neutron beam and circularly averaged to produce 1D scattering curves which can be combined to yield the full scattering profile^41^.

### LPNP technique

LPNP analysis was conducted using a Quadrasorb SI surface area and porosimetry analyzer. The samples were crushed to 20–35 mesh (0.50–0.85 mm), oven-dried at 110 °C under vacuum for 24 h to remove volatile substances and free water. Nitrogen adsorption/desorption isotherms were obtained at 77.3 K, through measuring the adsorption branch of nitrogen under the relative pressure P/P_o_ ranging from 0.010 to 0.995 and desorption branch from 0.995 to 0.010. The total pore volume and pore size distribution were determined using the BJH method^42^.

### MICP technique

The MICP technique can effectively determine connected porosity and pore-throat size distribution, using a mercury intrusion porosimeter (AutoPore IV 9510; Micromeritics Corporation)^5^. The cubic sample with a linear dimension of 10 mm was dried at 60 °C for at least 48 hours to remove moisture, and cooled to room temperature (~23 °C) in a desiccator with relative humidity less than 10%. Then, low- (5 to 30 psi; 0.034 to 0.21 MPa) and high-pressure (30 to 60000 psi; 0.21 to 413 MPa) analyses were initiated by progressively increasing the intrusion pressure while monitoring the volume change of mercury at a detection limit of <0.1 μL. Pore-throat size distributions from MICP tests were obtained using the Washburn equation^43^, with the confinement correction of contact angle and surface tension of mercury in shale nanopores^27^. The corrected pore-throat diameters cover a measurement range of 50 μm to 2.8 nm for the experimental conditions (e.g., using a penetrometers with a filling pressure of 5 psi) suitable for shale samples with porosities commonly less than 5%. Pore structure parameters (such as pore-throat size distribution, median pore-throat size, pore volume, pore area, and porosity) can be obtained for multiple connected pore networks at nm-μm spectrum^8^.

## Results

### SANS

#### Scattering length density (SLD) of shale samples

The SLD value is a measure of the scattering strength of a material component which depends on the scattering strength of the constituent scatterers (i.e. nuclear scattering length for neutrons) as well as their average volume density^44^. The porous rock samples can generally be treated as a two-phase system (solid matrix and pore space), where the number of scatterers and the difference of SLD controls the scattering intensity $I(Q)$ ^19,35^. The SLD for each mineral component in a porous medium can be obtained with the following formula:

1 $$SL{D}_{n}=\frac{{N}_{A}d}{M}\sum _{j}{p}_{j}{(\sum _{i}{s}_{i}{b}_{i})}_{j}$$

where *N*_*A*_ is Avogadro’s number (6.022 × 10^23^ mol^−1^), *d* is the grain density (g/cm^3^), *M* is the molecular weight of a mineral component (g/mol), *p*_*j*_ is the fraction of phase *j* within the material, *s*_*i*_ is the abundance of nucleus *i* in phase *j*, and *b*_*i*_ is the coherent scattering amplitude for nucleus *i* ^35^. It is generally accepted to use an average SLD calculation for the mineral matrix, including organic matter^45^. Average matrix SLD of rock sample can be calculated using the following formula^29^:

2 $$SL{D}_{rock}=\frac{{\sum }_{1}^{n}vol \% (k)SLD(k)}{100}$$

where *k* is a mineral component (including organic matter); *n* is the total number of components; SLD(k) is the SLD of mineral component *k*.

According to the XRD-derived mineral compositions (Table 2) and TOC contents (Table 1), average matrix SLD values calculated from Eq. 2 for four shale samples are presented in Table 1. The SLD of four samples displays a slight difference, with the order of Niobrara marl (4.21 × 10^−10^ cm^−2^) > Utica shale (3.92 × 10^−10^ cm^−2^) > Wolfcamp shale (3.71 × 10^−10^ cm^−2^) > Bakken shale (2.66 × 10^−10^ cm^−2^).

**Table 2:** Mineral composition in weight and volume percent of the studied samples.

Component	Density (g/cm^3^)	Niobrara		Wolfcamp		Bakken		Utica	
		wt.%	vol.%	wt.%	vol.%	wt.%	vol.%	wt.%	vol.%
Quartz	2.65	9.05	7.63	24.8	22.2	29.7	27.9	15.3	13.6
Orthoclase	2.56	/	/	/	/	23.4	22.8	/	/
Anorthite	2.73	3.02	2.55	2.99	2.60	2.89	2.63	/	/
Calcite	2.71	73.4	75.5	45.8	50.1	1.71	1.97	51.5	56.0
Dolomite	2.87	0.78	0.61	/	/	7.13	6.25	/	/
Gypsum	2.36	/	/	/	/	2.71	2.85	/	/
Illite	2.75	10.12	8.22	2.22	1.92	/	/	/	/
Chlorite	2.65	/	/	/	/	6.86	6.44	8.87	6.56
Muscovite	2.81	/	/	19.2	16.2	6.31	5.57	20.6	17.3
Marcasite	4.89	/	/	/	/	3.79	1.93	/	/
Pyrite	5.01	0.88	0.39	1.55	0.73	5.86	2.91	/	/
TOC	1.30	2.88	5.10	3.40	6.21	9.79	18.8	3.60	6.54

#### Analysis of the SANS data

**Figure 1:**
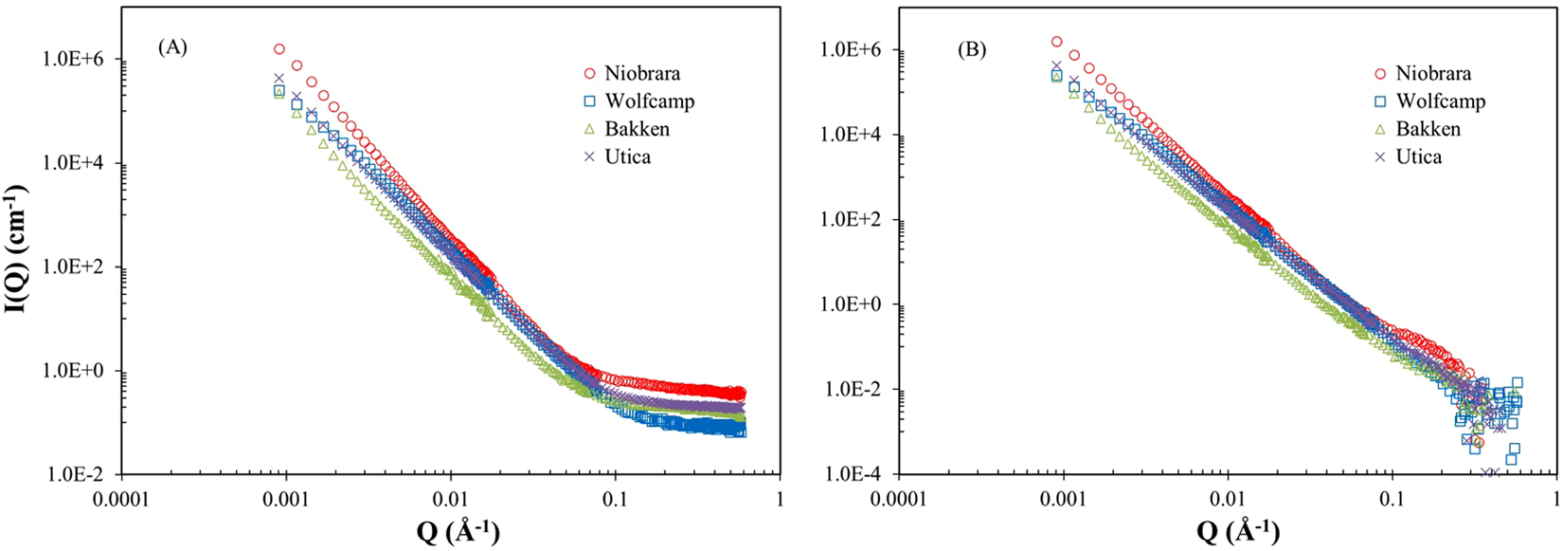
The SANS raw (**A**) and background subtracted (**B**) scattering profiles of the four samples.

As is commonly observed in complex heterogeneous systems like shale, log–log scattering profiles (i.e. *I(Q) vs. Q*) of the four studied samples exhibit a linear trend (i.e. power law scattering) in the low- and intermediate-*Q* range (<0.1 Å^−1^), and a gradual flattening of the curves towards a constant background scattering value at high-*Q* (Fig. 1A). This flat background can originate from the incoherent scattering of Hydrogen atoms in the organic matter and adsorbed water of shale, as well as the coherent scattering from pores <25 nm in the rock matrix^29,34,46^. The background values are obtained from the slope of the plot of *Q* ^4^ *I(Q) vs. Q* ^4^, where high- *Q* values dominate^47^. Niobrara marl exhibits background scattering that is substantially larger than those observed in the other samples. High-*Q* region of the scattering profiles shows a substantial change after performing background subtraction (Fig. 1B). In the meantime, the reliable data with a wide power-law distribution extend up to *Q* ≈ 0.25 Å^−1^ with a slope of the linear region ranging from 3.1 to 3.5 for four samples, which is related to surface fractal dimensions of *D*_*s*_ = 2.5 to *D*_*s*_ = 2.9^29^. As surface fractal dimension ranges from 2 (perfectly smooth surfaces) to 3 (extremely rough surfaces)^34^, the Wolfcamp, Bakken and Utica shales have slightly rough pore-matrix interface because their *D*_*s*_ values are close to 3 (2.8–2.9), while the Niobrara marl has a smaller surface fractal dimension (2.5) indicating a slightly smooth interface^46^. In other words, when the surface fractal dimension is up to 3, the pore-matrix interface is folded and almost completely fills the pore space. In fact, the high porosity Niobrara marl is dominated by mineral-associated pores to be angular and sharp edged, which reflects a slightly smooth pore-matrix interface^48^.

#### Porosity and pore-size distribution from SANS test

Neutron scattering profiles obtained from a polydisperse pore system are characterized by power law distribution (i.e. linear slope)^49^. The polydisperse spherical pore (PDSP) model has been widely applied to analyze total porosity and pore-size distribution in sedimentary rocks from SANS data^29,50^. The scattering intensity fitted by the PDSP model can be represented as^50^:

3 $$I(Q)=\frac{d\Sigma }{d\Omega }(Q)={({\rho }_{1}^{\ast }-{\rho }_{2}^{\ast })}^{2}\frac{\varphi }{{\bar{V}}_{r}}{\int }_{{R}_{min}}^{{R}_{max}}{{V}_{r}}^{2}f(r)P(Q)dr$$

where $\bar{V}$ is the average pore volume; ${\rho }_{1}^{\ast }$ and ${\rho }_{2}^{\ast }$ are the SLDs of - matrix (including organic matter) and pore space, respectively; $\varphi$ is the total porosity of the shale sample; R_max_ and R_min_ represent the maximum and minimum pore radius, respectively; $f(r)$ is the probability density of the power-law pore size distribution; and $P(Q)$ is a form factor of a sphere described by Eq. 4^29^:

4 $${F}_{sph}({Q}_{r})=9\,{[\frac{{\sin }(Qr)-Qrcos(Qr)}{{(Qr)}^{3}}]}^{2}$$

For all four samples, the PDSP model fits the linear region of the background-subtracted scattering profile (10^−3^ < *Q* < 0.25 Å^−1^), corresponding to pore radii ranging from 1 to 300 nm. The PDSP-model porosity for four samples shows an obvious difference (Table 3). With a value of 2.91%, the Niobrara marl displays the highest porosity, compared to 1.99% for Wolfcamp, 1.53% for Bakken and 1.48% for Utica shale samples.

**Table 3:** Porosity and cumulative pore volume of the studied samples obtained from different methods.

Sample ID	Porosity (%)					Cumulative pore volume (10^−2^ cm^3^/g)			
	PDSP	Porod^1^	Porod^2^	MICP^1^	MICP^2^	SANS	LPNP	MICP^1^	MICP^2^
Niobrara	10.66	15.5	12.5	4.36	4.23	4.35	1.41	1.78	1.73
Wolfcamp	4.89	7.3	6.0	0.51	0.47	1.91	0.54	0.20	0.19
Bakken	4.17	5.0	4.5	1.62	1.39	1.45	0.43	0.66	0.57
Utica	4.23	6.8	5.8	0.79	0.48	1.64	0.46	0.31	0.24

Porod^1^ porosity is calculated from the extrapolated range of Q on both ends of scattering profiles, while Porod^2^ porosity is from the Q range of 10^−3^ Å^−1^ to 0.25 Å^−1^.

MICP^1^ contains the cumulative pore volume for pore-throats ranging from 2.8 nm to 50 µm (a full range measurable for shale samples by MICP), as compared to MICP^2^ from 2.8 nm to 600 nm (measurable range of SANS analyses for comparison).

The model-independent Porod invariant method is also widely used to determine the total porosity of shale samples^45^. The porosity is estimated through the Porod invariant ${Q}_{{inv}}$ in a two-phase system^35,51^:

5 $${Q}_{inv}={\int }_{0}^{\infty }{Q}^{2}I(Q)dQ=2{\pi }^{2}{({\rho }_{1}^{\ast }-{\rho }_{2}^{\ast })}^{2}\varphi (1-\varphi )$$

The evaluation of ${Q}_{{inv}}$ is performed by three *Q*-domain integration: (a) unmeasured domain between 0 and *Q*_min_ (10^−3^ Å^−1^); (b) experimentally accessible domain *Q*_min_ < *Q* < *Q*_max_ = 0.5 Å^−1^; and (c) unmeasured *Q* > 0.5 Å^−1^. For direct comparison, we also present results based on Porod invariant using the same $I(Q)$ data with PDSP model. Total porosities obtained from the PDSP model and the Porod invariant show a slight difference, but a similar trend for the four shale samples (Table 3). Porod invariant porosity is systematically higher than that obtained from the PDSP model, which we attribute to the inclusion of extrapolated low- and high- *Q* values not considered in the PDSP analysis. In the same *Q* range, the PDSP porosity is slightly lower, but agrees well with Porod invariant porosity.

**Figure 2:**
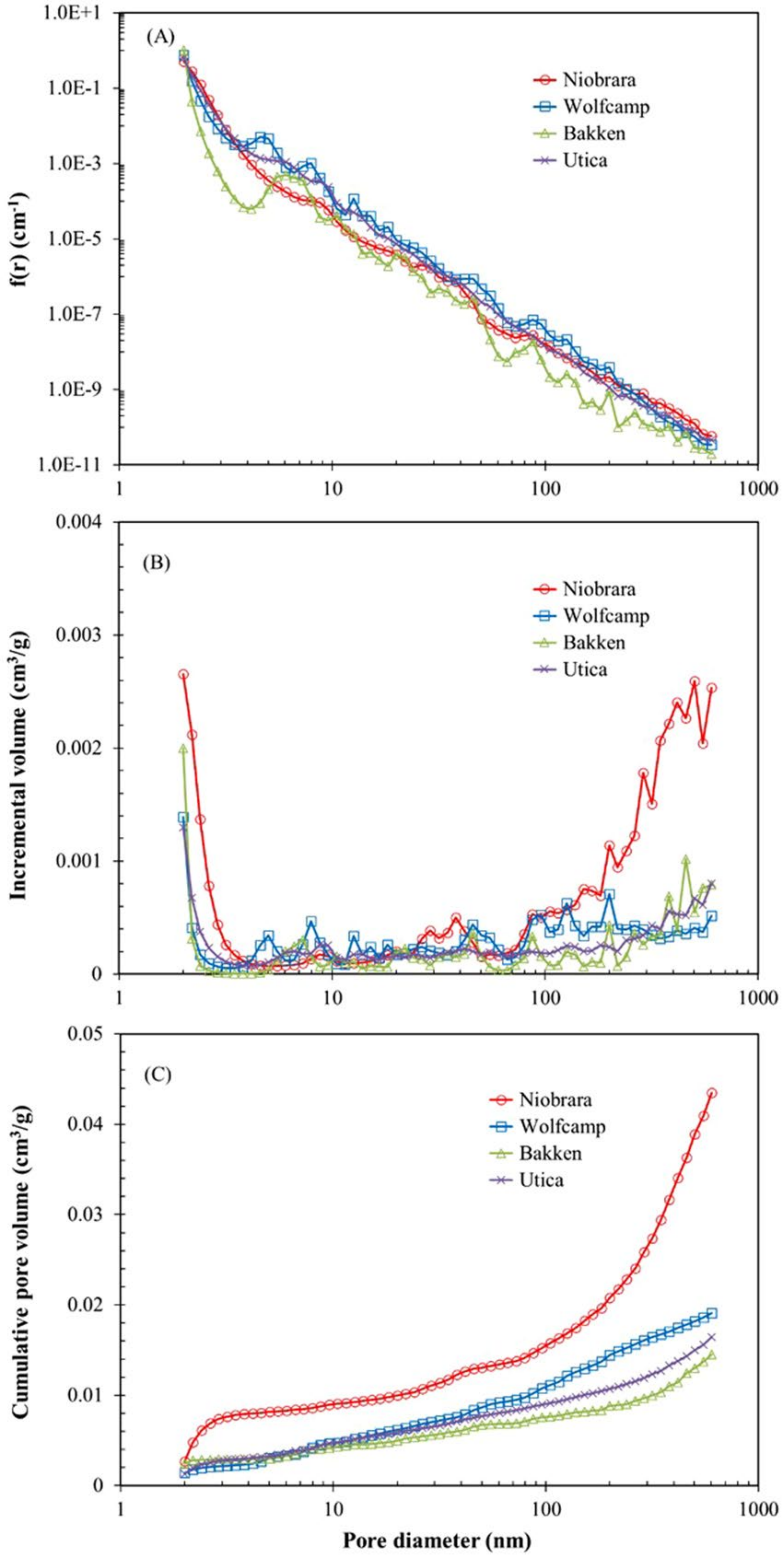
Plots of the probability density $f(r)$ (**A**) incremental pore volume (**B**) and cumulative pore volume (**C**) vs. pore-size diameter for the four samples using the PDSD model.

Neutron scattering obtained from polydisperse pore system is characterized by power law distribution^50,52^. Therefore, the PDSP model is reasonable to fit the linear portion of the scattering profile, where *Q* value ranges from 10^−3^ to 0.25 Å^−1^ in this study. Pore size distributions $f(r)$ are obtained from applying the PDSP model using PRINSAS software^49^ and are illustrated in Fig. 2A. The pore size distributions (ranging from 2 to 600 nm) follow a similar decreasing trend with an increase of pore size. In order to provide more direct information of the pore volume with respect to pore diameter, we convert the probability density of pore distributions $f(r)$ into pore volume distributions (Fig. 2B). All samples display a bimodal pattern of pore size distributions, with peaks at ∼2 nm and 460–600 nm of pore diameters. The Niobrara marl sample shows a high pore volume at pore diameters ranging in 2–4 nm and 30–600 nm. In contrast, the Bakken shale sample shows a relatively low pore volume in the entire range of SANS-measurable pore diameters (2–600 nm), except for a slightly high value for pores >380 nm.

The cumulative pore volume distributions are calculated by summing individual pore volumes and shown in Fig. 2C. The Niobrara marl exhibits the highest pore volume (4.35 × 10^−2^ cm^3^/g) and the Bakken shale sample the least (1.45 × 10^−2^ cm^3^/g), with the average pore volumes of four samples being 2.34 × 10^−2^ cm^3^/g (Table 3). For the Niobrara marl sample, the cumulative pore volume increases very rapidly at pore diameters smaller than 2.4 nm and larger than 200 nm, where the slope of the curve is much steeper than other pore regions. The cumulative pore volume of the Wolfcamp shale is higher than the Bakken and Utica shales at pore diameters larger than 8 nm and 13 nm, respectively. In additoion, the cumulative pore volume distributions of the Wolfcamp, Bakken and Utica shale samples are very close at pore diameters smaller than 20 nm (Fig. 2C), while the volume of Wolfcamp shale increases rapidly at pore diameters larger than 80 nm.

### LPNP

#### Isotherms of N_2_ adsorption and desorption

**Figure 3:**
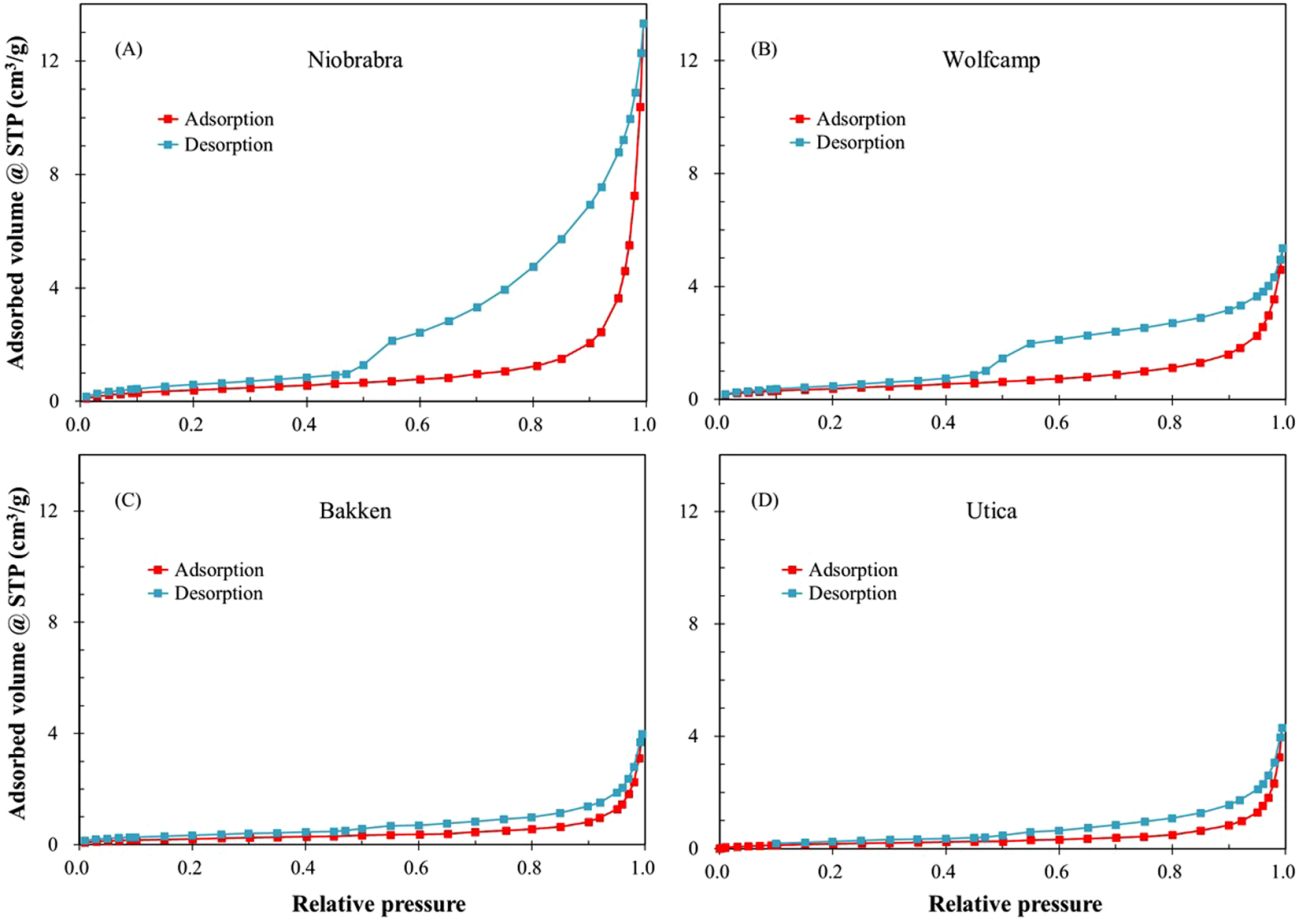
Nitrogen adsorption and desorption isotherms for the four shale samples at 77.3 K.

Nitrogen isotherms of adsorption and desorption for four shale samples are shown in Fig. 3. The adsorbed volume ranges from 3.98 to 13.3 cm^3^/g; in particular, the Niobrara marl show a much higher value than other samples. According to the IUPAC classification^53^, the N_2_ adsorption isotherms of four samples exhibit type IV adsorption, but without plateaus at high relative pressure regions. The adsorbed volume rises slowly at low relative pressure region until the relative pressure is close to 1.0, which indicates the presence of a certain number of both mesopores (2–50 nm) and macropores (>50 nm) in these shales. In addition, although the adsorbed volume is low, all isotherms show some adsorption at low relative pressure (*p/p*_*o*_ < 0.01) interval, relating to the presence of micropores (<2 nm). The ‘forced closure’ is present in the desorption branch at p/p_o_ about 0.45, which is related to the so-called ‘tensile strength effect’, which is attributed to an instability of the hemispherical meniscus in pores during the desorption stage^54^. All isotherms show a type H_3_ pattern of hysteresis loop, which is related to the presence of slit-shaped pores^26,53,55,56^. However, this interpretation may be subjected to errors based on the SEM and SANS analysis^2,26^.

#### Pore-size distribution by LPNP method

**Figure 4:**
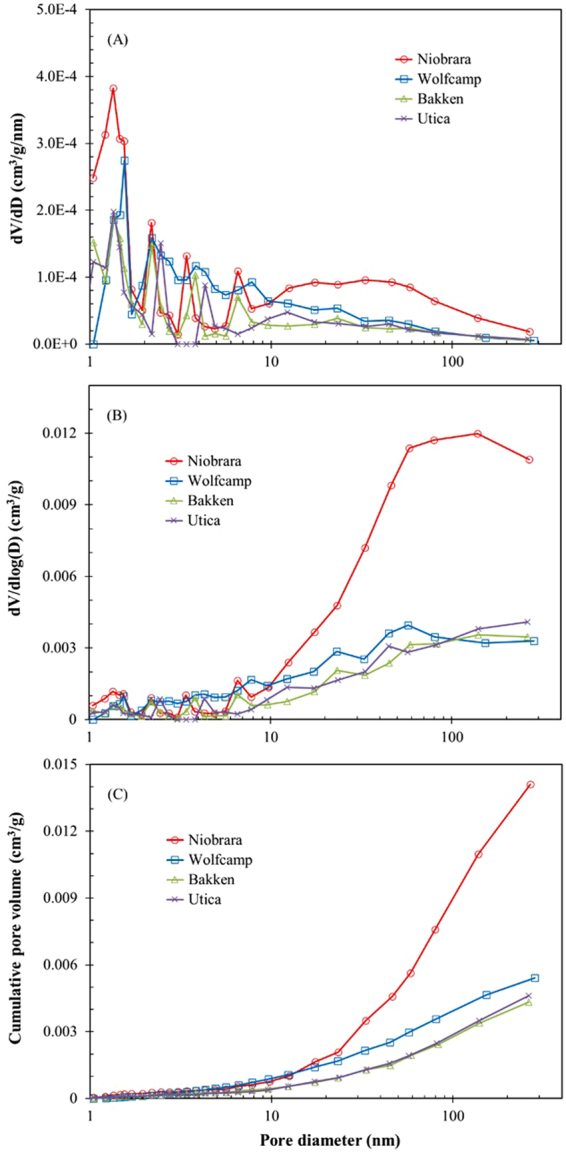
Plots of pore volume distributions (**A**,**B**) and cumulative pore volume (**C**) vs. pore-size diameter from the N_2_ adsorption of four samples using BJH model.

Figure 4 shows the results for pore-size distribution (*dV/dD vs. D* and *dV/dlog(D) vs. D*) and cumulative pore volume obtained with BJH model for the adsorption branch. The plot of *dV/dD vs. D* shows a broad pore size distribution (1–300 nm) with the peak value around 1.3–1.5 nm. The concentrations display a decreasing trend with an increase of pore size in all samples (Fig. 4A). The Niobrara marl shows another broad peak in pore sizes ranging from 20 to 60 nm, which is obviously different from other samples. The plot of *dV/dlog(D) vs. D* proportioned the real volumes reveals that pores sizes larger than 10 nm significantly contribute to the total pore volume (Fig. 4B). The contribution of pores sizes ranging from 1–2 nm and 10–300 nm in Niobrara marl sample shows the same trend with the plot of *dV/dD vs. D*, where the value is much higher than that of other three shale samples. The pore-size distribution of the Bakken and Utica shale samples shows a similar trend in both plots (Fig. 4A; B).

The cumulative pore volumes were determined from adsorption isotherms using the BJH model (Table 3, Fig. 4C). The Niobrara marl exhibits the highest pore volume (1.41 × 10^−2^ cm^3^/g), whilethe Bakken sample shows the least (0.43 × 10^−2^ cm^3^/g), with the average pore volumes of four samples being 0.71 × 10^−2^ cm^3^/g. Other than the Niobrara marl sample, these values quite agree with Barnett, Haynesville, and Eagle Ford shale samples analyzed by Clarkson *et al*. (2013)^3^. This work also shows that all samples have a large amount of pore volume in pore sizes larger than 10 nm, especially for the Niobrara marl sample.

### MICP

#### Mercury intrusion and extrusion curves

**Figure 5:**
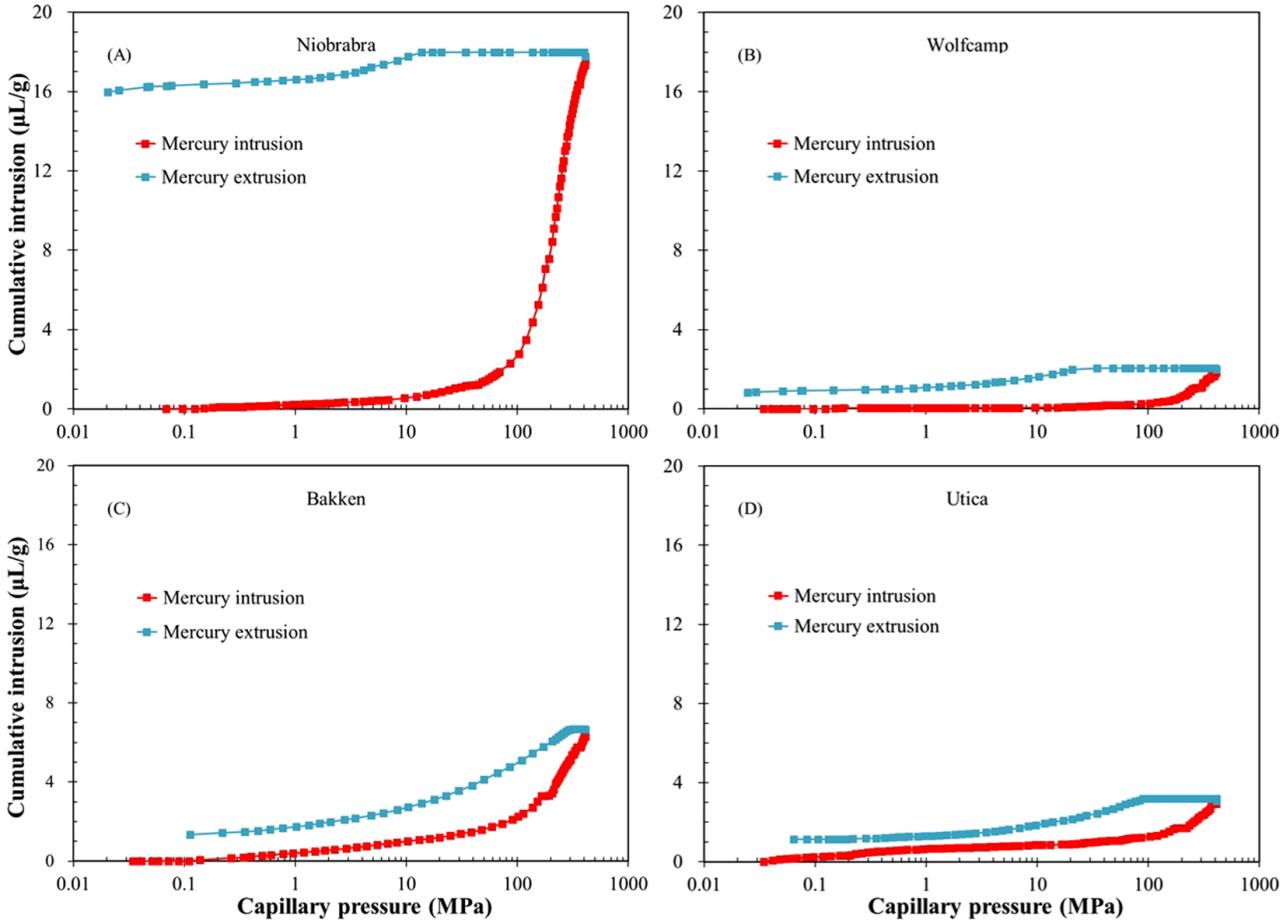
Mercury intrusion and extrusion curves for the four samples.

The plots of cumulative mercury intrusion and extrusion volume vs. the corresponding pressure for four shale samples are shown in Fig. 5. With an increase of intrusion pressure from 0.21 to 413 MPa (corresponding to pore-throats ranging from 50 µm to 2.8 nm), the intrusion volume of mercury gradually increases in all samples, indicating the presence of both mesopores and macropores. Specifically, the cumulative intrusion volumes at the highest pressure point show an obvious difference of Niobrara marl (17.8 µL/g) > Bakken shale (6.6 µL/g) > Utica shale (3.1 µL/g) > Wolfcamp shale (2.0 µL/g) (Table 3).

Similar to LPNP tests, hysteresis also occurs in mercury intrusion and extrusion cycles for all samples, indicating that about 20–90% of mercury remains trapped in samples after the pressure recovering to the initial one (Fig. 5). In general, during the extrusion from narrow pore necks, the snapping-off of the liquid meniscus would lead to a formation of the isolated droplet in the ink-bottle shaped pores^57^, which partly contributes to the entrapment of mercury.

#### Porosity and pore-throat distribution by MICP method

The total connected porosity of four shale samples shows an obvious difference, ranging from 0.51% to 4.36%, with the average value of 1.82% (Table 3). Such MICP porosity is the largest in the Niobrara marl sample and the smallest in the Wolfcamp shale sample.

**Figure 6:**
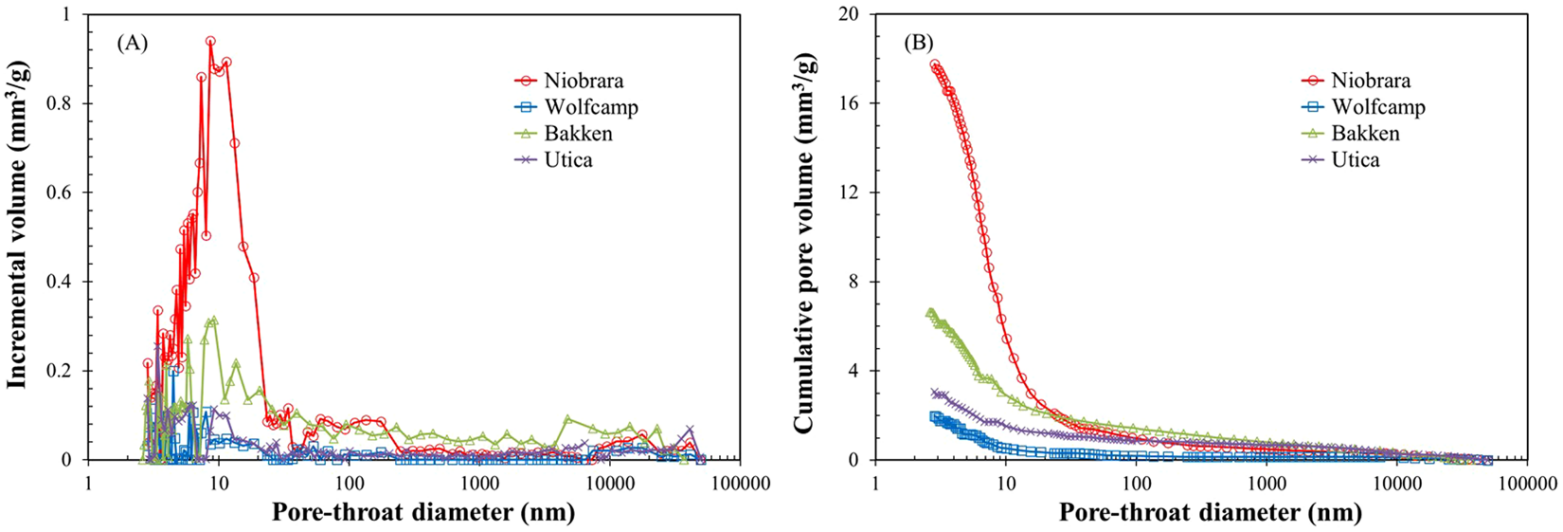
Plots of incremental pore volume vs. pore-throat diameter (**A**) and cumulative pore volume vs. pore-throat diameter for the four samples (**B**) using MICP method.

The pore-throat size distribution and cumulative pore volume results are shown in Fig. 6 for four shale samples. Pore-throat distribution shows a much broader range (2.8 nm–50 µm) than LPNP and SANS results. All samples display a unimodal pattern of pore size distributions with a broad peak between 3 and 10 nm in pore-throat diameters. There is a remarkable peak at 10 nm in pore-throat diameter for the Niobrara marl, which is much higher than other samples. Noticeably, the volumes of pores with a pore-throat diameters less than 50 nm dominate in all shale samples, with a proportion in the order of Niobrara marl (92.6%) > Wolfcamp shale (86.9%) > Bakken shale (73.7%) > Utica shale (67.5%). The results are consistent with previous studies on Barnett, Horn River, Longmaxi, Marcellus, Woodford, and Utica shales using both MICP and LPNP analyses^14,58–62^. In addition, from the plot of pore-throat diameter vs. cumulative pore volume, the total pore volumes of four samples exhibit an obvious difference, which is mainly ascribed to the volume of pores with pore-throats less than 20 nm (Fig. 6C).

## Discussion

### Comparison of SANS and fluid-invasion methods

#### Comparison of porosity and cumulative pore volume

The porosity and cumulative pore volume obtained from SANS and fluid-invasion methods show obvious differences. The values from SANS, especially obtained from Porod invariant analysis, are much higher than those from the MICP and LPNP methods (Table 3). In contrast, MICP method provides a wider pore-throat size ranging from 2.8 nm to 50 µm, while SANS and LPNP methods only detect pore features of 2–600 nm, and 1–300 nm, respectively. In order to provide more direct comparison, we also calculate the MICP porosity and cumulative pore volume in the pore-throat diameter range of 2.8–600 nm, and cumulative pore volume in the pore diameter range of 2–600 nm from the LPNP method, which are much lower than the SANS method (Table 3). This result is likely related to the ability of SANS to probe total pore volume, including both open (accessible) and closed (inaccessible) pores, while fluid-invasion methods can only examine pores accessible to corresponding fluid molecules under the conditions of the experiment^29,35^. From this consideration, we deduce that inaccessible pores contribute significantly to the total pore volume in the four shale samples. Inaccessible pores can occur as both intraparticle (within minerals and organic matter) and interparticle, and both could be widely developed in shale, coal, carbonate and siltstone^2,3,20,30,37^. We will specifically discuss the inaccessible porosity of these four American shale samples in next section. The pore volume at throat diameters of 2–2.8 nm is not measured by the MICP method, but pores of this interval are shown to contribute a significant portion of volume to the total porosity as measured using SANS and LPNP. There are similar porosity and cumulative pore volume distributions obtained from the SANS and LPNP methods, with an order of Niobrara marl > Wolfcamp shale > Utica shale > Bakken shale, and these show a discrepancy from the MICP results (Table 3). Based on MICP tests, although the Niobrara marl sample exhibits higher porosity and cumulative pore volume than the other samples, the Wolfcamp and Utica shale samples display lower values than the Bakken shale sample. There are at least two possible reasons for such a discrepancy. On one hand, the pore-throat size controls the intrusion of mercury into a connected pore network. According to the Washburn equation^43^ with the confinement correction for shale nanopores^27^, the pores with throats larger than 2.8 nm can be filled by mercury at the maximum pressure (413 MPa) achieved by the instrument. Figure 4 shows that a greater amount of pores with diameters less than 2.8 nm exists in the Wolfcamp shale compared to the Bakken shale. It is probable that there is a larger pore volume connected via pore throats less than 2.8 nm in the Wolfcamp shale than that in the Bakken shale, which causes the discrepancy of MICP with other methods. On the other hand, due to capillary resistance, effective pressures can be generated during MICP tests, which would result in pore collapse or crack closure^21,63^. Meanwhile, ductile minerals such as organic matter and clays in mudstone might also undergo an elastic deformation^64^. According to high-pressure Wood’s metal injection and Broad Ion Beam-SEM imaging results^21^, plastic deformation of the clay matrix leads to a cutting-off of pore pathways in the silt-rich Boom Clay. The Utica and Wolfcamp shale samples contain higher clay mineral contents than other samples (Table 2), which may cause the lower volume of mercury injection during the MICP test.

#### Comparison of pore-size distribution

**Figure 7:**
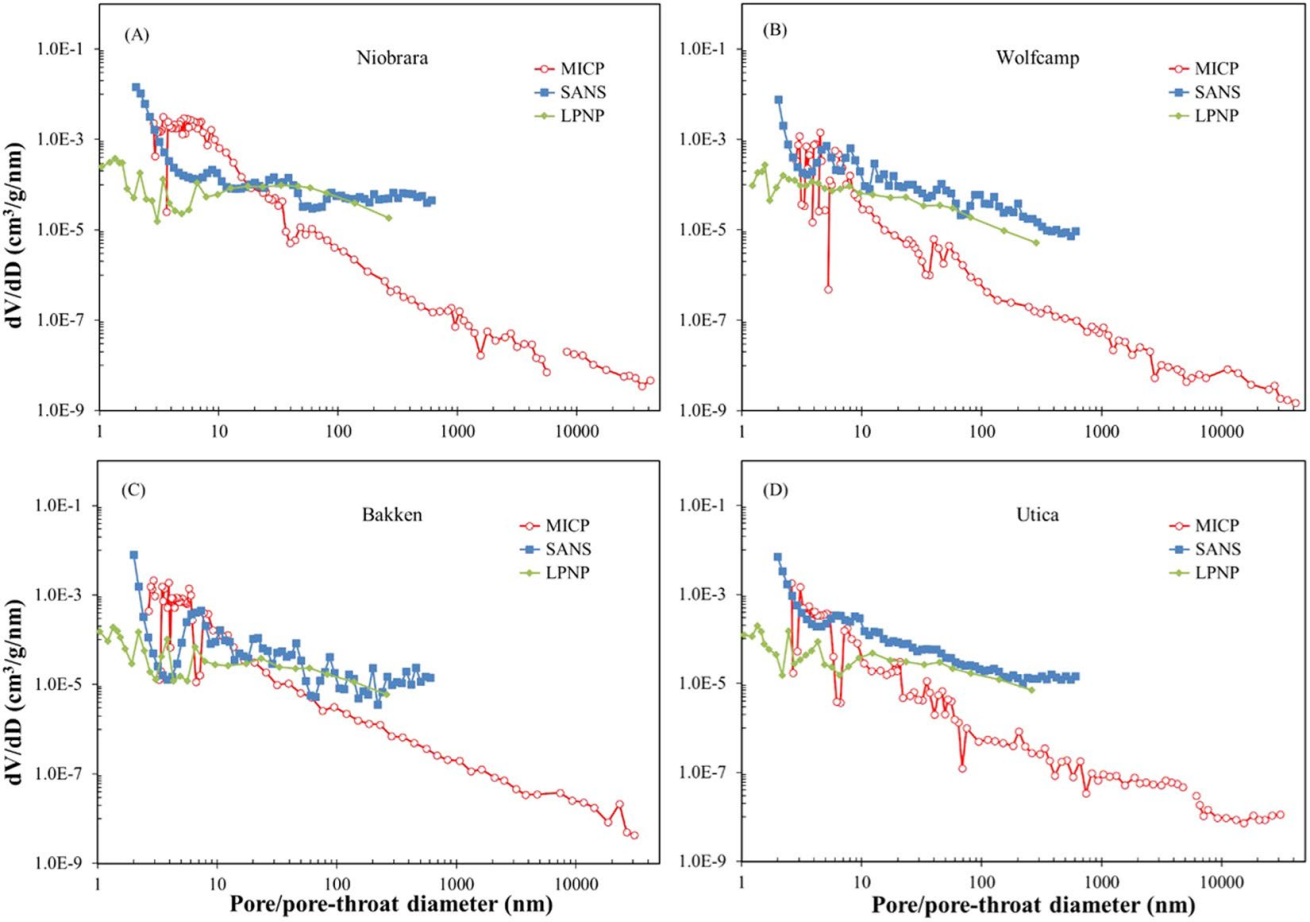
Combined SANS (PDSP), low-pressure N_2_ adsorption and MICP pore volume distributions with pore size for four samples.

To aid the comparison of the pore-size distribution obtained from SANS and from fluid-invasion methods, we unify the coordinate system (*dV/dD vs. D*), and compare the results with combined data from different methods (Fig. 7). The SANS and LPNP methods (both measure pore bodies) give comparable pore-size distributions in all samples, especially pore diameters larger than 10 nm, indicating a relatively good pore connectivity at this pore size range. On the contrary, the relatively large difference of pore size distributions between SANS and LPNP in the Wolfcamp and Utica shale samples indicates a poorly-connected pore system, which is also a possible reason to account for low porosity and cumulative pore volume obtained from the MICP test.

There is an obvious discrepancy between MICP results and other methods for all samples. The pore-size distribution of MICP results is higher than SANS and LPNP data for pore sizes <8–20 nm, beyond which the values decrease rapidly. More importantly, MICP measures is essentially a pore-throat distribution, while the SANS and LPNP methods quantify pore bodies. Therefore, the incremental volume of mercury at a given pressure represents the connective pore volume through a corresponding diameter of pore throat during MICP tests^21^. A number of publications have shown that MICP would overestimate the small pores and underestimate the large pores in various fine-grained materials, because of complicated pore shapes^64–66^. In fact, pore-throat distributions are strongly controlled by pore shapes. SEM observations show that many ink bottle-shaped pores are common in mudstones^11,67^, thus the larger volume of “bottle” pore bodies are counted within the “neck” pore throat size range during MICP analysis, which tends to skew the apparent pore size distribution.

**Figure 8:**
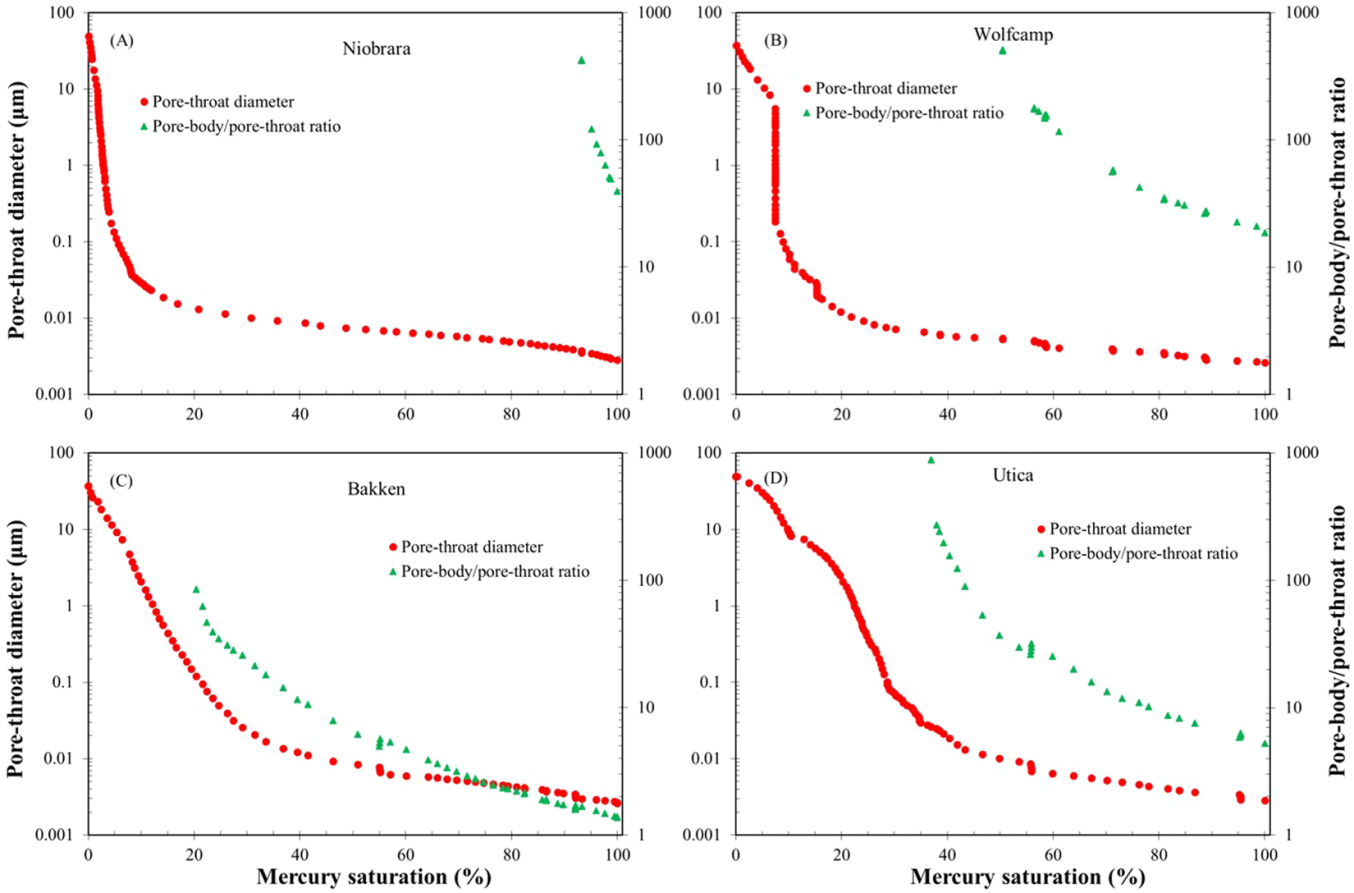
Plots of pore-throat diameter and pore-body/pore-throat ratio vs. mercury saturation for the four samples.

Measured hysteresis between the intrusion and extrusions curves can provide pore-body/pore-throat ratios^68^, which is indeed important to understand the pore shape information. The work of Anovitz and Cole (2015) on shale pointed out that with the decrease of the pore size, the size of pore-body gets close to pore-throat^69^. Figure 8 shows the MICP results of pore-body/pore-throat ratio for the four shale samples, which range from 1.4 to 420 with the 2.8–3.7 nm pore throat diameter interval (Fig. 8). Body-throat ratios generally decrease between samples from Niobrara marl (ranging in 39–420) > Wolfcamp shale (18–56) > Utica shale (5.2–7.5) > Bakken shale (1.4–1.9). The pore-body/pore-throat ratios of these four shale samples are generally consistent with Posidonia Shale (ranging from 1 to 2000 at 3 to 7.2 nm interval in pore-throat diameters) from the Hils area in Germany as reported by Klaver *et al*. (2012)^67^. The high pore-body/pore-throat ratio is the main factor to result in an overestimation of small pores and an underestimation of large pores for MICP method. The Niobrara marl shows a substantially higher pore-body/pore-throat ratio than other samples, leading to a larger difference of pore size distribution between MICP and other methods at pore throat diameters smaller than 10 nm (Fig. 7A). In addition, another possibility for the discrepancy of MICP results is that the compression of the samples at high intrusion pressures would lead the peak to move towards smaller pore size^70^, although compression has only a minor effect. These compressibility for these four shales from MICP tests range from 3.65 × 10^−5^ to 3.45 × 10^−4^ 1/Psi, and this is equivalent to a very small volume reduction.

### Determination of multi-scale closed (inaccessible) porosity

The connectivity of pores exerts a significant contribution to matrix permeability and gas diffusion in pores^71^. Closed porosity may be a key factor to control oil/gas storage, transport pathways, and production behavior^23,32^. SANS data provide the information of total porosity, while fluid-invasion methods give the connected porosity accessible from sample edge. Therefore, the fraction of closed porosity inaccessible to fluids such as N_2_ and mercury can be determined by comparing the difference between porosity obtained by SANS and fluid-invasion methods (Table 4). The total inaccessible porosity for mercury ranges from 52.3% to 89.1% for four samples at a pore-diameter interval of 2.8 to 600 nm; while for N_2_ ranges from 46.3% to 67.1% by LPNP data at an interval of 2 to 300 nm. The difference is attributed to the low value of porosity obtained from MICP. Inaccessible porosity in these four samples is slightly higher than Barnett shale with a reported inaccessibility of CD_4_ to be about 30%^28^, and Alberta Cretaceous Shale in Canada with a value of 20–37%^43^, but is close to 69.9% reported for over-mature Longmaxi carbonaceous shale in China^31^.

**Table 4:** Total and multiple-scale inaccessible porosity of the studied samples.

Sample ID	Total inaccessible porosity (%)		Multi-scale inaccessible porosity (%)				
	MICP/SANS	LPNP/SANS	2–5 nm	5–10 nm	10–50 nm	50–100 nm	100–300 nm
Niobrara marl	52.3	46.3	97.9	60.2	4.49	26.9	38.7
Wolfcamp shale	89.1	67.1	88.6	75.9	57.8	49.7	65.3
Bakken	50.2	56.4	95.2	84.2	56.4	13.3	5.59
Utica	82.8	62.1	96.5	90.5	57.9	37.9	22.7

**Figure 9:**
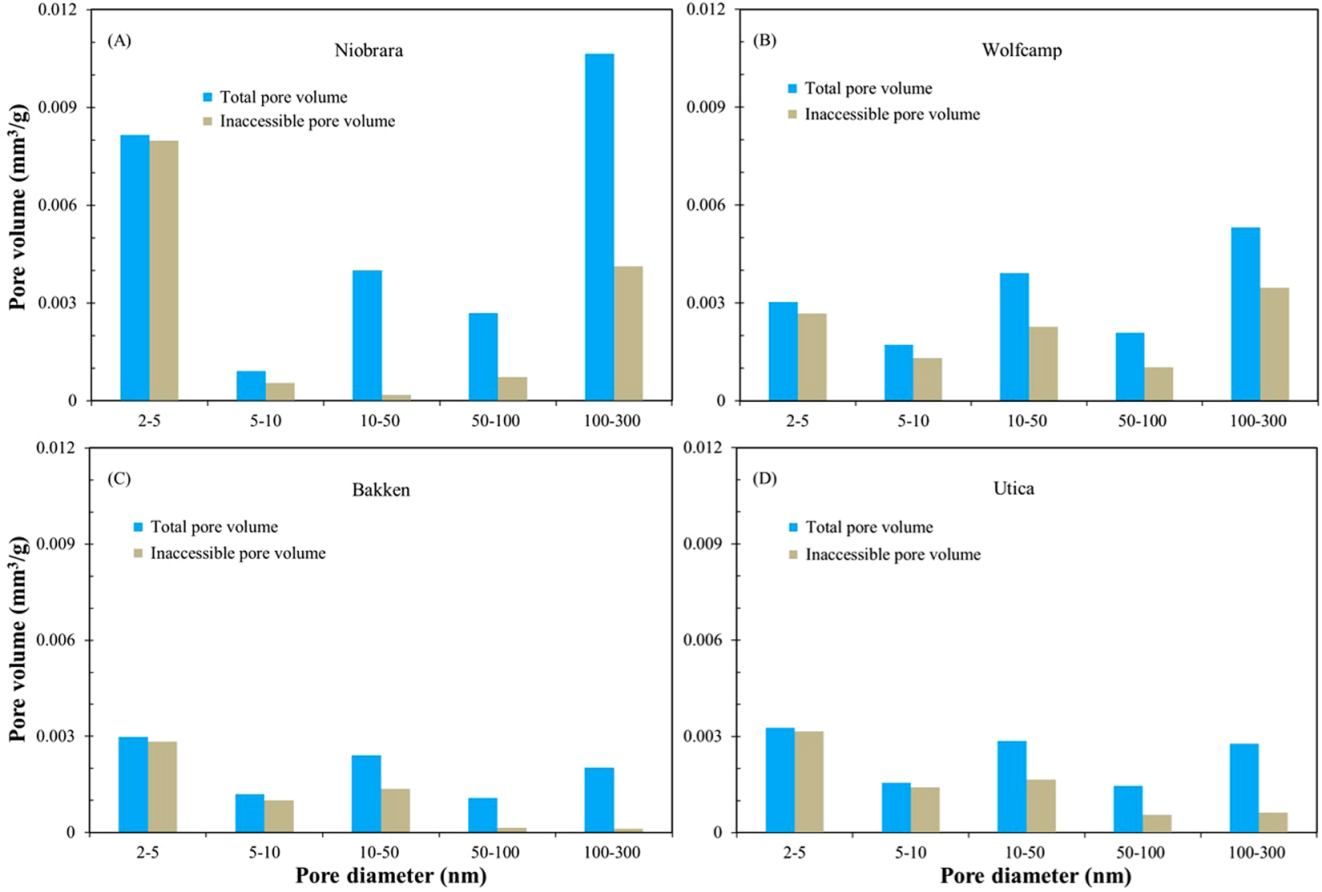
Plots of total and inaccessible pore volume vs. multiple-scale pore diameter intervals for the four samples using SANS and low-pressure N_2_ adsorption methods.

In order to investigate the relationship between the inaccessible porosity and pore size, we also calculate the multi-scale (five pore-diameter intervals) inaccessible porosity for N_2_ using SANS and LPNP methods, as they both measure pore bodies (Table 4). The inaccessible porosity of the four samples at different pore-diameter intervals shows different values and distributions (Fig. 9). Overall, the high inaccessible porosity occurs at pore diameters <10 nm, the value ranging from 60.2% to 97.9 with an average of 86.1%. At pore diameter range of 10–50 nm, the Wolfcamp, Bakken and Utica shale samples show a similar value of inaccessible porosity (56.4–57.9%); while the Niobrara marl exhibits a very low inaccessible porosity of 4.49%, where occurs a “overlap” region of the SANS and LPNP analysis (Fig. 7A). The distributions of inaccessible porosity for the Niobrara marl and Wolfcamp shale display a similar trend with an initial increase followed by a decrease, which is consistent with CD_4_ inaccessible porosity of Barnett shale^3^. In contrast, inaccessible porosity of Bakken and Utica shale samples decrease with the increasing pore diameter. Specifically, the volume of inaccessible pores in these two shale samples is low at the region of pore diameter > 100 nm, which explains the agreement of the corresponding pore volume distributions obtained from SANS and LPNP methods (Fig. 7C; D).

### Possible factors leading to closed porosity

Pore networks in shale are complex and controlled by the primary composition of grain assemblages, organic matter content, thermal maturation and burial diagenesis^11,72–76^. In this study, we preliminarily analyze the influencing factors leading to the closed porosity. Closed pores can develop within mineral and organic matter particles (as intra-particle pores) and between mineral particles or mineral and organic particles (as inter-particle pores). The pore volume in organic matter particles increases during thermal maturation, and large amounts of organic matter pores form in the post-mature stage^10,18^. Based on FIB-SEM observations, the organic particles in Posidonia shales (at R_o_ of 1.45%) from Hils area only contain small pore networks, which are not well connected with the surrounding mineral matrix^77^. The four samples in this study are all in the oil window with a similar maturity level (R_o_ ranging from 0.67% to 1.05%), indicating that organic matter pores should only just be developing^60,78–80^. We suggest that the lack of abundant pores in the organic matter results in low connectivity of organic pores. Kuila and Prasad (2011) also suggest that pores in clay samples with diameters less than 10 nm are associated with interlayer spaces^81^. In their BIB-SEM study of early mature Posidonia Shale from the Hils area in Germany, Klaver *et al*. (2012)^67^ indicated that large pores in fossils and calcite grains are connected through a low-permeable clay-rich matrix, which controls the connectivity of the matrix. Similar results were reported by Keller *et al*. (2011)^82^, who also found that connective porosity in Opalinus clay is just about 10–20% based on FIB-SEM technique.

According to the multi-scale inaccessible porosity data, the closed pores with diameters <50 nm (especially <10 nm) are dominant in the four shale samples. A large number of studies have shown that the diameters of organic matter-hosted and clay associated pores are mainly located in this range^10,11,14,83^. There is a significant correlation being observed between closed porosity and clay contents in the four shale samples. The Niobrara marl is dominated by calcite (peloids) with minor amounts of TOC and clay minerals. According to the work of Michaels (2014)^48^, the Niobrara Formation has undergone significant extents of diagenesis in the form of compaction, pressure-solution, and the subsequent reprecipitation of the pressure-solved calcite. However, all Michaels’ samples exhibited some amounts of intercrystalline pores associated with the calcite in peloids. The mineral-associated interparticle pores were the most abundant pore type within Niobrara marl samples^46^. Therefore, the Niobrara marl sample displays a low closed porosity, especially in pore diameters ranging from 5 to 10 nm.

With higher clay contents than the Niobrara sample, the Utica and Wolfcamp shalesdevelop a higher inaccessible porosity at pore diameters of <50 nm interval. Plastic deformation of the clay matrix my lead to a cutting-off of pore pathways during compaction, which can contribute to a significant volume of the closed porosity. A presence of pores with diameters of 2–5 nm in shales can be correlated with the dominance of the illite-smectite type of clays^84^. ‘Intra-tachoid’ pores (∼3 nm) are reported to be formed by a stacking of elementary unit cells in tachoids, which are building block of illite-smectite clays in rock-physics modelling of shales^84^. These incompressible 3 nm pores are hard to connect. Therefore, the high inaccessible porosity occurs at pore diameters ranging from 2 to 5 nm in all samples. With uniquely high TOC content and low maturity, the Bakken shale also displays a high closed porosity at pore diameters <50 nm interval. Based on the reported work of Liu *et al*. (2017)^85^ and our SEM observations, the organic matter pores are not well developed in the Bakken Shale, while inter- and intraparticle mineral pores are the dominant pore types. We interpret that abundant plastic organic matter, at 9.79% of TOC content, can clog the pore throat to lead to more isolated pores after compaction due to the lack of more solid mineral frameworks. Note that the SEM imaging of organic-rich shales can only resolve pores with sizes larger than 5 nm, while pores in 2 nm are near the instrument’s resolution^20^. The absence of imaged pores of pores with diameters of 2–5 nm suggests that there is still an ambiguous structure within either the mineral or organic matter.

### Evaluation of methods used for pore structure analyses

Pore structure plays a significant influence on reservoir quality, oil/gas contents and fluid properties in shale^15,58,76,86,87^. The unique property of shale pore structure obtained from different measurement methods indicates that the accuracy of pore structure measurements may be problematic, although the individual measurement techniques are sound^88^. The discrepancy of results from different methods is partly related to the different detection ranges of the pore diameters, space (pore body or pore throat), pore type (open or closed pores), theoretical bases and data deduction models, but a single factor cannot thoroughly explain all of the differences.

For the SANS methods, the intensity of scattered neutrons is highly sensitive to choice of the average SLD value for the rock matrix. Error in the measured porosity and pore-size distributions could result from core-scale shale heterogeneity leading to inaccurate SLD calculation. It is generally accepted to use an average SLD calculation for the mineral matrix, but the SLD likely varies with pore size^28^, due to the size dependence of both the geometry and associated pore wall material of shale porosity. For example, large pores mainly occur between mineral particles, and small pores are commonly developed in organic matter particles in shale, so both minerals and organic matter contribute to the significant value to SLD. In addition, high-density minerals, such as pyrite, could have a strong effect on calculated SLD^45^. However, pyrite only contributes 0 and 3.6% to the bulk mineralogy in our samples, and its influence can be excluded.

One of the major uncertainties of the LPNP method is the influence of sample crushing. Primary structure and fabric will change at the microscopic level, which inevitably changes the surface properties of samples and may alter the original pore structure or generate new pore space^89–91^. During a crushing process, compression and shear forces acting on the samples also generate smaller fragments and induce fracture propagation as well^92^. In addition, no unified standardization of grain size is used for LPNP tests raises the question about direct data comparisons among different laboratories^93^. In general, the use of smaller shale particles would increase measured micro- and meso-pore volumes by enhancing pore accessibility according to Han *et al*. (2016) and Wei *et al*. (2016)^94,95^, who studied the sample sizes from 0.113 to 4 mm and from 0.075 to 0.25 nm, respectively.

Sample size is probably a major contributor to the observed difference among methods in this work, especially for the MICP method in the use of 10-mm sized cubic sample, while SANS (150-μm thick thin section) and LPNP (500–850 μm) methods use a similar, and much smaller, sample size. The work of Hu *et al*. (2012; 2015)^5,96^ indicates the distribution of edge-accessible connected pore spaces in rock, and the sample-size dependent pore connectivity is more pronounced for fine-grained shale. To further assess the sample size effect on pore accessibility, we are currently measuring both bulk and particle densities, and MICP porosities for a range of rock with a wide range of pore connectivity, at multiple sample sizes.

The SANS method examines the total porosity and cannot give the information of connective pores. However, these interconnections, namely pore throats, are of critical importance to oil/gas transport. Although the SANS and LPNP methods provide comparable pore-size distributions, they cannot distinguish pore bodies from pore throats. The MICP measurements provide a direct information of pore throats, though it results in some discrepancies of pore size distribution results noted between MICP and other methods. The high hydrostatic pressures during MICP tests likely leads to inelastic deformation via compaction of shale samples^63^. According to the work of Penumadu and Dean (2000)^97^, the compaction volume can reach up to 20% in kaolin clay. For validating the MICP method, we have utilized several approaches such as runs without solid samples and with impervious samples. In addition, we have been periodically (monthly) analyzing a standard sample (soft alumina silica) with a mono-modal pore size of 7 nm. The results show that no changes of pore-throat size are observed with the same test protocol as for shale samples (e.g., pressures up to 60000 psi), suggesting that the material compression effect is very small using our developed MICP method.

With the awareness of limitations associated with each method, the combined methodology of each complementary technique serves as an effective way to understand the pore structure of shales, and calculate the multiple-scale inaccessible porosity.

## Conclusions

1. SANS is an effective measure to determining total porosity and pore-size distribution in shale with a strong presence of nm-sized pore spaces.
2. The pore structure of four typical American shale formations obtained from SANS and fluid-invasion methods shows an obvious difference, though SANS and LPNP methods give comparable pore-size distributions considering their similarity in sample sizes and measurement of pore bodies. The discrepancy between MICP results and those from SANS and LPNP methods is attributed to characterization of pore-throat distribution.
3. Multiple-scale (five pore-diameter intervals) inaccessible porosities for N_2_ are determined using SANS and LPNP data. Overall, the high inaccessible porosity (ranging from 60.2% to 97.9% with average of 86.1%) in four shale samples occurs at pore diameters <10 nm, which we attribute to isolated organic matter-hosted and clay-associated pores.
